# Transforming Practical Veterinary Anatomy Teaching (2013–2026): From Demonstration to Team-Based Learning

**DOI:** 10.3390/vetsci13101124

**Published:** 2026-10-21

**Authors:** Nieves Martín-Alguacil, Luis Avedillo, Rubén A. Mota-Blanco, Mercedes Marañón-Almendros, Miguel Gallego-Agundez

**Affiliations:** 1 Departmental Section of Anatomy and Embryology, Faculty of Veterinary Medicine, Universidad Complutense de Madrid, 28040 Madrid, Spain; luiavedi@ucm.es (L.A.); mdemaran@ucm.es (M.M.-A.); migugallt@ucm.es (M.G.-A.); 2 Veterinary Department, Faculty of Biomedical and Health Sciences, Universidad Europea de Madrid, 28040 Madrid, Spain; ruben.mota@universidadeuropea.es

**Keywords:** academic performance, active learning, anatomy education, collaborative learning, curriculum development, higher-order thinking, student-centered learning, veterinary education

## Abstract

This study evaluated the long-term effects of transitioning from traditional practical teaching to Team-Based Learning (TBL) in veterinary anatomy education. A longitudinal analysis was conducted across thirteen student cohorts at the Faculty of Veterinary Medicine at the Complutense University of Madrid between the academic years 2013/14 and 2025/26. The transformation of the curriculum gradually replaced instructor-centered practical sessions with a structured TBL model incorporating readiness assurance tests, team application exercises, independent study and supplementary digital resources. Student performance was evaluated using practical examination scores, individual and team readiness assurance tests (iRATs and tRATs) and application-oriented cognitive assessments (AOCAs). Sectional anatomy performance improved from scores below 5.0 during the traditional teaching phase to above 6.5 in recent cohorts, with lower variability in scores. Meanwhile, osteology grades increased from 4.33 ± 2.54 in 2019/20 to 5.06 ± 1.96 in 2025/26, and AOCA scores rose from 4.51 ± 2.70 to 8.20 ± 1.50. Team readiness scores consistently exceeded individual scores, highlighting the value of collaborative learning. Independent study and engagement with digital resources were associated with significantly better outcomes, particularly in AOCAs. Overall, these results suggest that TBL should be integrated into veterinary anatomy curricula in the long term due to its positive impact on academic performance, collaboration, and higher-order cognitive skills.

## Simple Summary

This longitudinal study examined how practical veterinary anatomy teaching evolved at the Faculty of Veterinary Medicine at the Universidad Complutense de Madrid over a 13-year period. The progressive implementation of Team-Based Learning (TBL), alongside independent study opportunities and digital learning resources, was found to be associated with improvements in student achievement, collaborative learning, and higher-order cognitive performance. Students who engaged more actively with independent study and digital resources generally achieved higher scores. These findings suggest that a structured, student-centered educational approach may contribute to favorable learning outcomes in veterinary anatomy.

## 1. Introduction

Over the past decade, veterinary education has undergone a progressive shift toward active, student-centered learning methodologies designed to enhance not only content acquisition but also the development of professional competencies [1,2,3,4,5,6,7]. Within this context, Team-Based Learning (TBL) has emerged as a structured, evidence-based pedagogical approach that promotes individual accountability, collaborative problem-solving, and the application of knowledge to authentic scenarios [8,9]. Its adoption across the health sciences has been driven by the need to prepare graduates capable of integrating complex information, communicating effectively, and working efficiently in multidisciplinary environments [10,11,12,13,14].

Traditionally, veterinary anatomy has been taught through a variety of approaches, including demonstrations and practical sessions based on prosection and dissection [15,16,17]. These methods are highly effective in developing anatomical understanding, fostering collaborative learning and problem-solving. However, some instructional formats place greater emphasis on identifying and recalling anatomical structures than on collaborative, structured reasoning and applying knowledge in authentic contexts [18]. Recognizing these limitations, our teaching team initiated a long-term transformation of the practical curriculum beginning in the 2015/16 academic year. This process culminated in the systematic implementation of TBL starting in 2019, initially in the practical sessions involving anatomical sections of the canine and equine heads. Over subsequent years, the methodology was progressively refined and extended to additional content areas. Throughout this period, we collected comprehensive data on individual and team performance, formative assessments, application exercises, and student and faculty perceptions. This longitudinal dataset provides a unique opportunity to evaluate the sustained impact of TBL on learning outcomes and engagement within a core preclinical discipline. The introduction of TBL represented a major pedagogical shift, expanding the focus of assessment and instruction beyond memorization toward competencies such as teamwork, communication, critical thinking, and applied anatomical reasoning [13,19].

This study evaluated the long-term educational outcomes associated with the progressive transformation of practical veterinary anatomy teaching from a traditional demonstration-based approach to a TBL model between 2013 and 2026. As well as describing the implementation process, the study examined changes in academic performance, collaborative learning outcomes and higher-order cognitive achievement throughout successive phases of curricular development. Particular attention was given to the evolution of individual and team preparedness, performance in osteology and sectional anatomy, and the contribution of complementary educational strategies, such as independent study activities and digital learning materials. It was hypothesized that the progressive implementation and refinement of TBL, together with these additional learning resources, would be associated with improved academic achievement, enhanced collaborative performance and greater development of higher-order cognitive skills among veterinary anatomy students.

## 2. Materials and Methods

### 2.1. Study Context and Setting

This longitudinal observational action-research study was designed to document and evaluate an ongoing curricular transformation implemented as part of routine educational development within the Veterinary Anatomy and Embryology curriculum. It examined educational outcomes across thirteen successive cohorts of students enrolled between the 2013/14 and 2025/26 academic years. As the curricular changes were introduced progressively as part of normal teaching practice, it was neither feasible nor educationally appropriate to randomly allocate students to different instructional approaches. Therefore, the findings should be interpreted as evidence derived from a longitudinal observational evaluation of a curricular transformation, rather than from an experimental investigation designed to establish causal relationships.

### 2.2. Participants

Participants were first-year veterinary students enrolled in the compulsory anatomy course each year. Cohort sizes ranged from 155 to 208 students across the study period. All students participated as part of the standard curriculum, and no additional recruitment procedures were required.

### 2.3. Ethical Considerations

The study analyzed educational outcomes obtained as part of routine curricular activities. All data were anonymized prior to analysis, and participation in course activities formed part of the standard curriculum. The study complied with institutional policies governing educational evaluation and quality-improvement activities that did not require formal ethical approval.

### 2.4. Instructional Materials

Across the study period, the following materials were used:

- Osteological specimens of the canine and equine skull (articulated and disarticulated) and sectioned skull in different spatial planes.
- Transverse, sagittal, and dorsal anatomical sections of the canine and equine head.
- Instructor-prepared worksheets.
- Readiness Assurance Tests (RATs) and team application-oriented cognitive assessment (AOCA) (from 2019 onward).
- Digital resources, including radiographic, MRI and CT images of the head (introduced progressively from 2016).

### 2.5. Study Design

This study was designed as an observational, longitudinal action research study to document and evaluate an ongoing curricular transformation implemented as part of routine educational development. As the intervention was implemented as part of routine curricular development, it was not feasible to allocate students randomly to instructional conditions. Therefore, this study should be interpreted as a longitudinal observational evaluation of a curricular transformation, rather than an experimental assessment of the effectiveness of TBL.

#### 2.5.1. Phase 1: Traditional Practical Teaching (2013–2015)

Practical sessions followed a conventional structure:

- Instructor-led demonstrations of osteological landmarks and sectional anatomy.
- Individual structure identification tasks.
- Minimal structured peer interaction.
- No formal pre-class preparation.

These sessions served as the baseline for comparison.

#### 2.5.2. Phase 2: Incremental Pedagogical Enhancements (2015/16–2018/19)

Beginning in 2015/16, the teaching team introduced targeted modifications aimed at increasing engagement and higher-order reasoning:

- Guided peer-discussion prompts.
- Short case-based questions linking head anatomy to clinical scenarios.
- Structured observation tasks for anatomical sections.
- Pilot pre-class reading assignments.

These enhancements informed the design of the subsequent TBL implementation.

#### 2.5.3. Phase 3: Systematic Implementation of TBL (2019–2026)

In 2019, TBL was formally integrated into practical sessions on head osteology and sectional anatomy. During the study period, the implementation of TBL varied between practical domains. TBL was continuously applied to sectional anatomy activities from 2019/20 onwards, but TBL-based osteology instruction was only delivered in the 2019/20, 2020/21, 2024/25 and 2025/26 academic years. Consequently, longitudinal osteology analyses were restricted to these cohorts, whereas sectional anatomy analyses included all cohorts throughout the study period. The TBL format included the following.

#### 2.5.4. Pre-Class Preparation

This includes assigned readings, digital modules, and labelled images of the head.

#### 2.5.5. Readiness Assurance Process

- Individual Readiness Assurance Tests (iRATs)
- Team Readiness Assurance Tests (tRATs) Teams of 5–7 students worked toward consensus on conceptual questions.

The readiness assurance tests (RAT) varied slightly throughout the osteology practical sequence, in line with the learning objectives of each session. In Osteology 1, which was the first module in which students were exposed to skull osteology, the iRAT included questions based on drawings and schematic representations of cranial bones, which helped them to acquire fundamental anatomical concepts and terminology. By contrast, the iRATs in Osteology 2 and 3 were based on images of real bone specimens, requiring students to identify anatomical structures in a more authentic context. The tRAT covered the same content as the corresponding iRAT and was completed by student teams following individual assessment [12]. This progressive approach was designed to facilitate the transition from recognizing basic anatomical features to interpreting real osteological structures.

#### 2.5.6. Application-Oriented Cognitive Assessment (AOCA)

The questions were designed to assess the application, analysis and interpretation of anatomy in clinical situations, rather than factual recall [20]. After completing the practical activities as part of the readiness assurance process, students undertook collaborative AOCA. This was designed to evaluate how they applied and integrated anatomical knowledge acquired during each practical session. Unlike the iRAT, which assessed pre-class preparation and factual knowledge, the AOCA focused on higher-order learning outcomes.

The assessment required students to identify anatomical structures in novel contexts, interpret spatial relationships, apply osteological knowledge to practical situations and solve anatomy-based reasoning problems. These assessments were aligned with the application and analysis levels of Bloom’s revised taxonomy, evaluating students’ ability to apply knowledge gained from individual preparation and team discussions to new anatomical problem [20].

Group discussions were held on AOCA, which were then scored on a 10-point scale. The format and level of difficulty were kept consistent across cohorts to allow for longitudinal comparison of learning outcomes. Scores obtained in these assessments were subsequently used as indicators of higher-order cognitive performance throughout the curricular transformation.

In the osteology sequence, AOCA were administered at the end of each practical session. These included questions requiring the recognition and interpretation of osteological structures, the identification of anatomical relationships, and the application of anatomical knowledge to clinically relevant scenarios. This approach enabled the evaluation of both factual knowledge acquisition and students’ ability to integrate and apply anatomical concepts in authentic learning contexts.

#### 2.5.7. Team AOCA

Teams solved clinically oriented problems involving:

- Interpretation of head sections.
- Identification of osteological landmarks relevant to surgical approaches.
- Integration of anatomical knowledge with imaging modalities.

Exercises followed the TBL “4S” principles: significant problem, same problem, specific choice, simultaneous reporting [21].

#### 2.5.8. Instructor Facilitation

Instructors acted as facilitators, guiding discussion and clarifying misconceptions.

The study included 13 successive student cohorts enrolled between the 2013/14 and 2025/26 academic years. Throughout this period, practical teaching evolved from a traditional instructor-centered format to a fully implemented TBL curriculum. Cohort characteristics and pedagogical phases are summarized in Table 1.

Table 1 shows the study cohorts included in the longitudinal analysis and the corresponding pedagogical phases. The study documents the transition from traditional instructor-centered practical teaching (2013/14–2014/15) through a period of progressive pedagogical enhancement (2015/16–2018/19) to the systematic implementation of TBL from 2019/20 onwards.

#### 2.5.9. Independent Study

Independent study sessions consisted of voluntary, instructor-led activities conducted outside of scheduled practical classes. During these sessions, students reviewed osteological specimens, discussed anatomical queries and received formative feedback. Attendance was recorded and subsequently used to compare the academic outcomes of participants and non-participants.

#### 2.5.10. Educational Context and Curriculum Environment

Throughout the study period, the veterinary curriculum remained structured as a five-year degree programme. Although the composition of the student cohorts inevitably differed from year to year, the course content, learning objectives and assessment framework for practical anatomy remained broadly comparable. Several educational developments occurred during the study period, including the implementation of Team-Based Learning and the progressive incorporation of digital learning resources, increased use of online learning platforms, independent study opportunities and wider adoption of educational technologies within higher education. The integration of digital tools and online learning resources was also accelerated by the subsequent arrival of the COVID-19 pandemic. Consequently, the educational context evolved over time, so some changes in student performance observed may reflect broader educational developments in addition to the curricular transformation described in this study.

Therefore, the present study evaluates the implementation of TBL within a dynamic educational environment rather than in isolation. The findings should be interpreted as reflecting a broader educational ecosystem that progressively incorporated collaborative learning strategies, independent study opportunities, and digital educational resources.

#### 2.5.11. Time-on-Task and Learning Activities

Throughout all pedagogical phases, the number of scheduled teaching hours devoted to head osteology and sectional anatomy remained largely stable (Table 2). However, the nature of student engagement changed progressively throughout the intervention. During the traditional teaching phase, learning was primarily based on instructor-led demonstrations carried out during scheduled laboratory sessions. During the transitional phase, practical sessions incorporated guided peer discussion, preparatory activities and clinically oriented questions. Following the implementation of TBL, students were expected to undertake structured pre-class preparation using assigned readings, digital resources, and learning materials. In addition, voluntary independent study sessions were developed to support autonomous learning. Consequently, the total amount of student engagement with anatomical content increased over time. This should be considered when interpreting longitudinal changes in performance.

#### 2.5.12. Assessment Consistency Across Cohorts

Throughout the study period, practical teaching and assessment were aligned with core learning outcomes related to osteology and sectional anatomy. While individual examination items and learning activities were periodically updated to reflect curriculum development and educational refinement, the assessments continued to evaluate comparable anatomical knowledge and identification skills, as well as spatial reasoning and the application of anatomical concepts. The assessments were designed and supervised by members of the academic unit responsible for teaching the course.

However, formal psychometric evaluation of the assessment instruments, including longitudinal measures of test and inter-rater reliability, was not systematically collected throughout the entire 13-year study period. Therefore, these evaluations could not be conducted retrospectively. This limitation should be considered when interpreting changes in student performance across cohorts.

#### 2.5.13. Data Collection

To evaluate the impact of the curricular transformation, the following data were collected:

- Student performance on practical assessments (structure identification and applied anatomy questions).
- RAT scores, including individual (iRAT) and team (tRAT) outcomes from 2019 onward.
- AOCA outcomes obtained during osteology and sectional anatomy practical sessions.

#### 2.5.14. Data Analysis

All statistical analyses were executed using **jamovi** (Version 2.7.38) (The jamovi project, 2026; Sydney, Australia) https://www.jamovi.org/. Although the data were not normally distributed, descriptive statistics are reported as mean ± SD to facilitate longitudinal comparisons across large cohorts, and to maintain consistency with previous educational research. Non-parametric tests were used for inferential analyses. Team-Based Learning outcomes. Prior to inferential analysis, the normality of the quantitative variables was assessed using the Shapiro–Wilk test and visual inspection of Q–Q plots. As several variables showed significant deviations from normality, non-parametric statistical procedures were employed for the inferential analyses. Differences between cohorts were evaluated using the Kruskal–Wallis test, followed by the Dwass–Steel–Critchlow–Fligner and Dunn post hoc procedures, with Bonferroni adjustment where appropriate. Effect sizes were estimated using epsilon-squared (ε^2^). Statistical significance was established at *p* < 0.05. Spearman’s rank correlation coefficient was used to evaluate the relationship between osteology achievement and sectional anatomy performance because of the non-normal distribution of the variables.

#### 2.5.15. Appendix

Detailed cohort-level data supporting the longitudinal analyses are provided in Appendix A, Appendix B, Appendix C and Appendix D. To enhance transparency and facilitate interpretation of the longitudinal analyses, detailed cohort-level outcomes are provided. Appendix A presents practical-session performance data for iRATs, tRATs, and AOCA across osteology and sectional anatomy cohorts. Appendix B presents longitudinal grade distributions for osteology and sectional anatomy practical sessions based on iRATs scores, allowing comparison of achievement patterns and score dispersion across cohorts. Appendix C contains detailed summary statistics for the 2024/25 cohort, including overall performance indicators, practical-session outcomes, independent study analyses, and achievement-category comparisons. These supplementary data provide the cohort-level information underlying several analyses presented in the Results Ssection. Appendix D contains detailed summary statistics for the 2025/26 cohort, including overall osteology performance outcomes, practical-session analyses, independent study and H5P resource engagement, and comparisons with the 2024/25 cohort.

These appendices provide detailed data underlying the analyses presented in the main manuscript and support replication of the educational intervention in other veterinary anatomy programs.

## 3. Results

A total of 2345 students distributed across 13 academic cohorts were included in the longitudinal analysis (Table 1). The study encompassed three pedagogical phases: traditional teaching (2013/14–2014/15), incremental pedagogical enhancement (2015/16–2018/19), and full implementation of TBL from 2019/20 onward. Figure 1 illustrates a typical TBL session for osteology.

### 3.1. Longitudinal Evolution Sectional Anatomy Performance

Descriptive statistics for all sectional anatomy cohorts are presented in Table 3, and the longitudinal trends are illustrated in Figure 2.

Student performance in sectional anatomy remained relatively low during the traditional and transitional phases, with mean scores ranging from 3.11 ± 1.99 to 4.88 ± 2.75. Following the implementation of TBL, however, average scores generally increased across cohorts, reaching 5.78 ± 1.39 in 2021/22, 6.55 ± 0.97 in 2024/25 and 6.67 ± 0.95 in 2025/26 (see Table 3).

The first two TBL cohorts (2019/20 and 2020/21) showed only modest improvement compared to previous years. However, a more consistent increase was observed from 2021/22 onwards. Additionally, score variability decreased in the most recent cohorts, indicating greater consistency in learning outcomes.

Overall, the curricular transformation was associated with higher performance in sectional anatomy and more consistent achievement across successive cohorts (Figure 2).

### 3.2. Longitudinal Evolution of Osteology Performance

Descriptive statistics for osteology outcomes are summarized in Table 4. As TBL-based osteology teaching was only implemented in the 2019/20, 2020/21, 2024/25 and 2025/26 academic years, these cohorts were the only ones included in the osteology longitudinal analysis.

Table 4 shows longitudinal osteology outcomes during the implementation of Team-Based Learning (TBL). Values are presented as cohort means and standard deviations (SDs) for the overall grade, as well as for the iRAT, tRAT and AOCA. The table illustrates the significant enhancement in osteology performance and higher-order cognitive outcomes that occurred after the TBL methodology was consolidated.

Longitudinal osteology outcomes are presented in Figure 3.

Overall, osteology grades increased substantially between the early implementation and consolidated TBL phases. The mean overall grade increased from 4.19 ± 1.63 in 2019/20, to 3.16 ± 0.54 in 2020/21, to 6.52 ± 0.76 in 2024/25, and finally to 6.78 ± 0.68 in 2025/26.

Different patterns emerged when the outcomes of the individual, team and AOCA were examined separately. Individual readiness (iRAT) scores remained relatively stable across cohorts, ranging from 4.33 to 5.40. In contrast, team readiness (tRAT) scores consistently exceeded individual scores, with mean values ranging from 6.18 to 7.20.

The greatest improvement over time was seen in AOCA, which increased from 4.51 ± 2.70 in 2019/20 to 7.60 ± 2.31 in 2020/21, 7.90 ± 1.41 in 2024/25 and 8.20 ± 1.50 in 2025/26.

These findings suggest that the curricular transformation had a greater impact on higher-order cognitive performance than on individual readiness measures.

### 3.3. Performance Across Osteology Practical Sessions

Performance varied across the osteology practical sessions (see Figure 4), with tRAT scores consistently exceeding iRAT scores in all cohorts.

In the 2020/21 cohort, both iRAT and tRAT scores decreased from Osteology 1 to Osteology 2, remaining similar in Osteology 3. Conversely, AOCA scores increased, peaking in Osteology 3. The 2024/25 cohort produced the lowest scores in all assessment domains in Osteology 2, followed by improvement in Osteology 3. The 2025/26 cohort demonstrated greater stability, featuring a modest increase in iRAT scores, relatively consistent tRAT performance and a decline in AOCA scores during Osteology 2, with partial recovery thereafter.

Across all cohorts, Osteology 2 was consistently associated with lower iRAT and tRAT scores than the other practical sessions, indicating greater difficulty. In contrast, AOCA outcomes varied between cohorts. Overall, students appeared able to adapt to the increasing cognitive and anatomical demands of the osteology sequence.

### 3.4. Relationship Between Osteology and Sectional Anatomy Achievement

A Spearman rank correlation analysis was performed using individual student scores from the 2024/25 cohort to evaluate the relationship between performance in osteology and sectional anatomy. A significant positive correlation was observed between overall osteology achievement and sectional anatomy performance (Spearman’s rho = 0.296, *p* < 0.001), indicating that students who achieved higher scores in osteology also tended to obtain higher scores in sectional anatomy.

While the strength of the association was moderate, these results imply that competencies acquired through osteology learning, such as acquiring anatomical knowledge, spatial reasoning, and interpreting anatomical structures, could enhance performance in sectional anatomy activities. These results support the integration of osteology and sectional anatomy learning within the veterinary curriculum.

However, the observed association should not be interpreted as evidence of a causal relationship. Rather, they suggest that achievement in both subjects may reflect a common set of anatomical skills and learning behaviors.

### 3.5. Comparison Between the 2024/25 and 2025/26 Cohorts

Comparisons between the two most recent cohorts revealed a significant increase in individual readiness performance. Mean iRAT scores rose from 4.45 ± 2.21 in 2024/25 to 5.06 ± 1.96 in 2025/26 (*p* = 0.0003). However, tRAT remained stable, with no significant differences detected between cohorts (5.76 ± 2.57 versus 6.00 ± 1.97; *p* = 0.188).

Performance in the second practical assessment improved significantly in 2024/25 (7.92 ± 1.23 versus 7.67 ± 1.11; *p* = 0.008), whereas no significant differences were identified for the first practical assessment. AOCA scores were slightly lower in 2025/26, though overall performance remained high.

Overall, these results suggest that the refinements introduced during the latest implementation phase were associated with improved individual preparation while maintaining strong collaborative performance.

### 3.6. Impact of Independent Study Participation

The effects of participating in independent study are summarized in Figure 5.

Students who participated in these activities achieved significantly higher scores than non-participants across all outcome measures (*p* < 0.001), including overall grade (7.12 ± 1.24 vs. 5.34 ± 1.58), iRAT (5.85 ± 1.82 vs. 3.91 ± 1.95), tRAT (7.65 ± 1.41 vs. 6.88 ± 1.63) and AOCA performance (7.88 ± 1.15 vs. 6.21 ± 1.48).

Effect sizes ranged from small to moderate, with the largest observed for AOCA performance (η^2^ = 0.117) and the smallest for team readiness performance (η^2^ = 0.031). Overall, these findings suggest that taking part in independent study activities is linked to better academic performance, especially in higher-order cognitive outcomes and personal preparation.

### 3.7. Combined Effects of Independent Study Participation and H5P Engagement

Figure 6 summarizes the combined effects of independent study participation and H5P use on AOCA performance. Mean scores remained above 7.4 for all preparation strategies. Students who participated in independent study activities and/or used H5P resources generally achieved the highest AOCA scores. However, students who relied solely on standard PowerPoint-based study guides also performed satisfactorily.

As only aggregated subgroup data was available, the results are presented descriptively and no inferential comparisons were made. Overall, the findings suggest that participation in independent study and engagement with H5P may support higher cognitive performance during the osteology sequence.

Supplementary analyses are provided in Appendix A, Appendix B, Appendix C and Appendix D. Appendix A presents cohort-level performance data for iRATs, tRATs and AOCA across individual practical sessions. Appendix B examines changes in readiness performance and score variability over time during the curricular transformation. Appendix C and Appendix D provide detailed descriptive statistics for the 2024/25 and 2025/26 cohorts respectively. These include practical-session outcomes, analyses of independent study and H5P engagement, and relationships between performance in osteology and sectional anatomy. Together, these supplementary data support and contextualize the longitudinal trends reported in the main manuscript.

## 4. Discussion

The present study documents a longitudinal transformation of the veterinary anatomy practical curriculum over a period of more than a decade. The findings suggest that introducing a TBL-centered curriculum alongside other educational innovations was associated with progressive improvements in academic performance, collaborative learning outcomes and higher-order cognitive achievement [22,23,24]. Collectively, these findings suggest that TBL may contribute positively to a broader instructional framework for teaching complex anatomical content to large groups of veterinary students. Although the study design does not allow for causal inference, the consistency of the observed trends across successive cohorts suggests that the curricular transformation was accompanied by favorable educational outcomes.

A key finding was the progressive improvement observed in sectional anatomy performance following the implementation of TBL. During the traditional teaching phase, student achievement remained relatively low and displayed considerable variability among cohorts. In contrast, the introduction and subsequent refinement of TBL were accompanied by higher mean scores and a gradual reduction in score dispersion. This pattern is consistent with improved academic outcomes and reduced variability in learning outcomes among students. The reduction in variability is particularly important in anatomy education, where large differences in prior preparation and spatial reasoning abilities often contribute to heterogeneous performance [25,26].

A similar trend was observed across all the cohorts in which TBL-based osteology instruction was implemented. While improvements were observed in multiple areas, the largest improvements were observed in AOCA rather than individual readiness activities. While individual preparation scores remained relatively stable throughout the study period, AOCA performance increased markedly across cohorts. This suggests that participation in TBL-based activities is more strongly associated with knowledge application and integration than with factual recall alone [19,27,28]. Interpreting structures, solving anatomically based problems and connecting osteological concepts with clinical scenarios are central objectives of veterinary anatomy education. The present findings are consistent with previous reports describing positive associations between TBL and higher-order learning processes [14,20,27,29,30,31].

The consistent difference observed between individual and team performance provides additional support for the educational value of collaborative learning. Across all cohorts and practical sessions, team readiness scores consistently outstripped individual ones. This suggests that peer discussion and collective decision-making are associated with more accurate responses than those achieved independently. These observations align with the theoretical principles of TBL, which propose that collaborative reasoning helps learners to recognize misconceptions, consider alternative interpretations, and facilitate conceptual understanding of the subject matter [32]. Notably, the persistence of this pattern across years indicates that the collaborative advantage is a consistent feature of the educational model rather than a phenomenon specific to a particular cohort.

Progressive improvements across cohorts may also be associated with an increase in the instructor’s experience of designing and implementing TBL activities. Effective TBL requires expertise in creating application-oriented tasks, leading group discussions and managing classroom dynamics. Consequently, any improvements observed during the later stages of implementation may reflect student adaptation to the instructional approach, as well as the maturation of teaching practice. As the present study was not designed to distinguish between these effects, it is not possible to determine their respective contributions.

Analyzing performance across individual practical sessions provided additional insights into the learning process. Practice 2 was found to be the most challenging stage of the osteology sequence, particularly in terms of individual preparedness and team performance. The lower performance observed in Practical Session 2 may be explained by several factors, including the change in instructional format. In addition to involving greater anatomical complexity, this session involved a transition from the schematic illustrations used in Practical Session 1 to identifying structures on real bone images. This requires students to transfer their previously acquired anatomical knowledge from simplified representations to authentic anatomical specimens, which demands greater visual–spatial processing. Visual–spatial ability has been consistently associated with learning and assessment performance in anatomy [33,34]. Furthermore, learning anatomy from real specimens requires learners to mentally manipulate and recognize three-dimensional structures from different perspectives, which is associated with greater cognitive demand than schematic representations [35]. Consequently, the decline observed in Practical Session 2 may reflect the increased visual–spatial cognitive load associated with this transition, rather than deficiencies in the teaching approach. The subsequent improvement in later sessions suggests that students adapted to these demands over time, which is consistent with evidence indicating that anatomy education can enhance visual–spatial abilities [36,37,38]. While the iRAT in Practice 1 was based on drawings and schematic representations designed to introduce first-year students to skull anatomy, the iRATs in Practices 2 and 3 required participants to identify structures on images of real bones. This demanded greater visual recognition and spatial interpretation skills. Nevertheless, AOCA scores remained comparatively high and generally improved in subsequent sessions. This suggests that, despite temporary declines in preparatory performance, students were able to adapt to increasing conceptual and visuospatial demands while maintaining progression in anatomical learning [7,19,21,39,40].

Students who participated in independent study activities achieved higher scores in all assessed areas. However, these findings should be interpreted with caution given that participation was voluntary. It is possible that students who were more motivated, engaged in their studies, or conscientious in general were also more likely to attend independent study sessions. Consequently, the observed association may reflect both the educational value of the activity itself and pre-existing differences among students. Nevertheless, the consistent pattern observed across multiple outcome measures suggests that structured independent learning opportunities may complement TBL.

The results summarized in Table 5 demonstrate that independent study participation influenced multiple dimensions of learning. While improvements were observed for overall grades and individual preparation, the largest observed effect size was detected in AOCA outcomes. This finding suggests that structured independent learning opportunities may be particularly effective in promoting knowledge integration and application, which represent key objectives of contemporary veterinary anatomy education [7,28,31,41,42].

The findings related to supplementary learning resources warrant consideration. Students who engaged in independent study activities, H5P resources, or both tended to achieve the highest scores across most practical sessions. However, students classified as not participating in independent study activities and not using H5P resources were not unprepared for practical sessions. Rather, all students had access to the standard instructional materials provided for each practical activity, including instructor-developed PowerPoint-based study guides, and were expected to prepare independently before attending laboratory sessions. Consequently, the comparisons presented in Figure 5 represent differences among preparation strategies rather than differences between prepared and unprepared students.

From this perspective, the relatively small differences observed among groups provide useful insight. AOCA scores remained high across all preparation modalities, suggesting that the standard instructional materials provided an effective foundation for student learning. Nevertheless, students who engaged in independent study activities, H5P resources, or both tended to achieve the highest scores across most practical sessions, indicating that these supplementary learning strategies may provide incremental benefits beyond conventional self-directed study. Importantly, these findings suggest that the educational gains associated with TBL were not dependent on a single instructional component. Rather, learning outcomes appear to have been supported by a complementary instructional framework combining structured team-based activities, independent preparation, traditional study materials, and digital learning resources.

Previous studies have reported on student perceptions of the TBL-based curriculum and demonstrated an increasing acceptance of active-learning methodologies over time [43,44]. The present study builds on these findings by providing objective evidence of improvements in academic performance and cognitive outcomes during the period in which TBL and related educational innovations were implemented [21]. From a broader educational perspective, the results have significant implications for the instruction of veterinary anatomy. Practical anatomy courses often encounter difficulties due to large student numbers, limited teaching time, and the complexity of three-dimensional anatomical content. These findings suggest that a structured TBL approach may help mitigate some of these challenges by encouraging active participation, collaborative problem solving and application-oriented learning [8,9,10,11,14,26,27,28,29,30,31]. The improvements observed across multiple cohorts further indicate that these educational benefits can be sustained over time within routine curricular practice.

Several limitations should be considered when interpreting the findings. Firstly, the study was conducted at a single veterinary institution, which may restrict how widely the results can be generalized to other educational contexts. Secondly, the longitudinal design involved successive student cohorts rather than randomized allocation to instructional conditions. Consequently, the improvements observed over time cannot be specifically attributed to TBL, as changes in cohort characteristics and other curricular developments may also have influenced student outcomes. Thirdly, multiple educational innovations were introduced progressively during the study period, including independent study activities and digital resources. This makes it impossible to determine the individual contribution of each component. The study compared cohorts enrolled over a 13-year period. During this time, changes in student characteristics, digital literacy, educational expectations and external educational circumstances may have affected learning outcomes independently of the curricular intervention. Therefore, the observed findings should be interpreted as reflecting the impact of a broader set of educational interventions rather than the isolated effect of TBL. A categorical approach was used to examine the relationship between independent study and academic performance, to facilitate educational interpretation. However, future studies could complement this analysis by using correlation or regression models to provide a more detailed evaluation of the associations between study habits and academic outcomes. In addition, formal measures of assessment reliability and inter-rater consistency were not systematically collected throughout the entire study period. This prevented a retrospective evaluation of measurement stability across cohorts. Furthermore, it was not possible to separate the influence of increasing instructor expertise in the design and delivery of TBL from the effects of the curricular transformation itself.

Overall, the results indicate a progressive transformation in the teaching of practical anatomy over the study period. The transition from instructor-centered demonstrations to a curriculum incorporating structured TBL was accompanied by improved academic achievement, reduced performance variability, stronger collaborative outcomes and higher AOCA scores. Although individual readiness scores remained relatively stable over time, team-based activities and application-oriented exercises were consistently associated with higher levels of achievement. These findings suggest that TBL could be a valuable component of a comprehensive, student-centered approach to first-year veterinary anatomy education.

## 5. Conclusions

In conclusion, the present study found that progressively implementing a TBL-centered curriculum alongside complementary educational innovations was associated with improvements in academic achievement and cognitive performance in veterinary anatomy practical sessions. The combination of collaborative learning, structured preparation, independent study opportunities and supplementary digital resources produced favorable educational outcomes across multiple cohorts. These results imply that TBL could be a valuable part of modern veterinary anatomy education. However, more research using controlled and multicenter study designs is needed to determine the exact impact of TBL and other elements of the educational system.

## Figures and Tables

**Figure 1:**
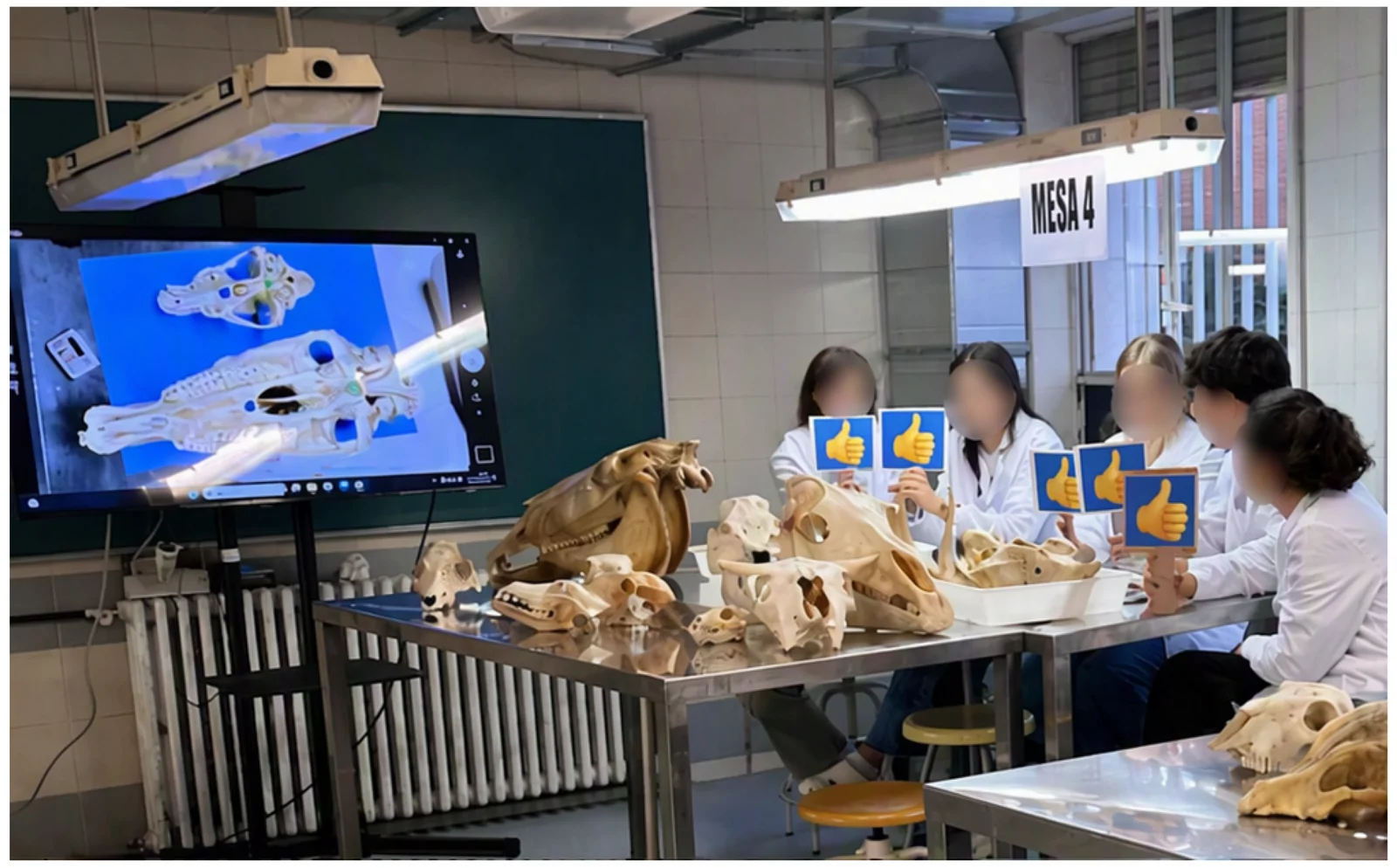
The collaborative correction of iRAT and tRAT activities during an osteology practical session. Student groups used a camera-based projection system to review questions on the identification of anatomical features on real bone specimens. Answers were indicated using colored modelling clay, enabling all group members to express their agreement or disagreement with the proposed response. Any differences between groups were resolved through discussion and justification of anatomical reasoning before consensus was reached.

**Figure 2:**
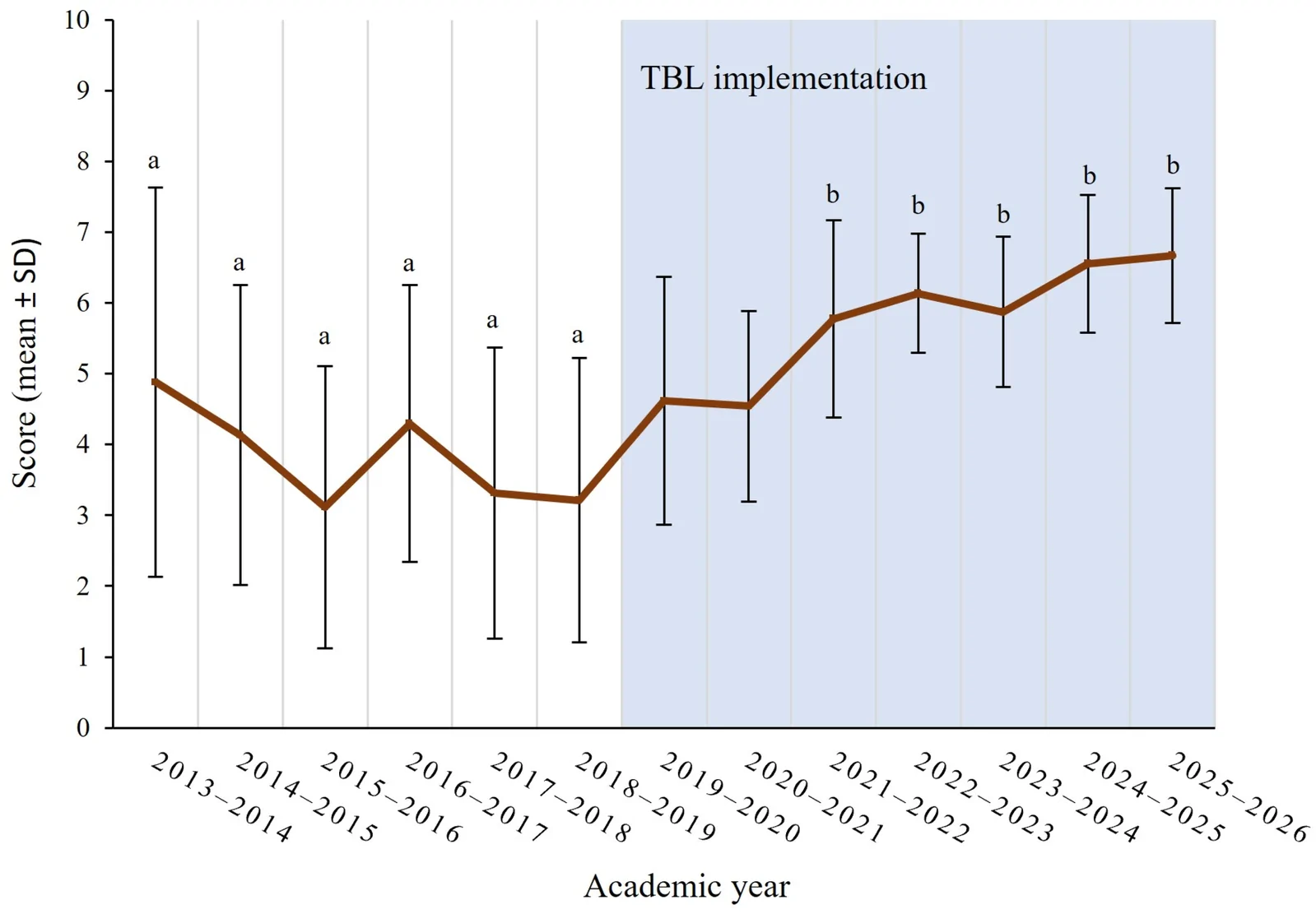
Longitudinal evolution of student performance in sectional head anatomy from 2013/14 to 2025/26. Values are presented as mean ± SD. The dashed vertical line indicates the introduction of Team-Based Learning (TBL) in 2019/20. Mean scores increased progressively following implementation of TBL, while score variability decreased over time. Different superscript letters indicate significant differences between the traditional teaching period (^a^) and the consolidated TBL period (^b^) (*p* < 0.05).

**Figure 3:**
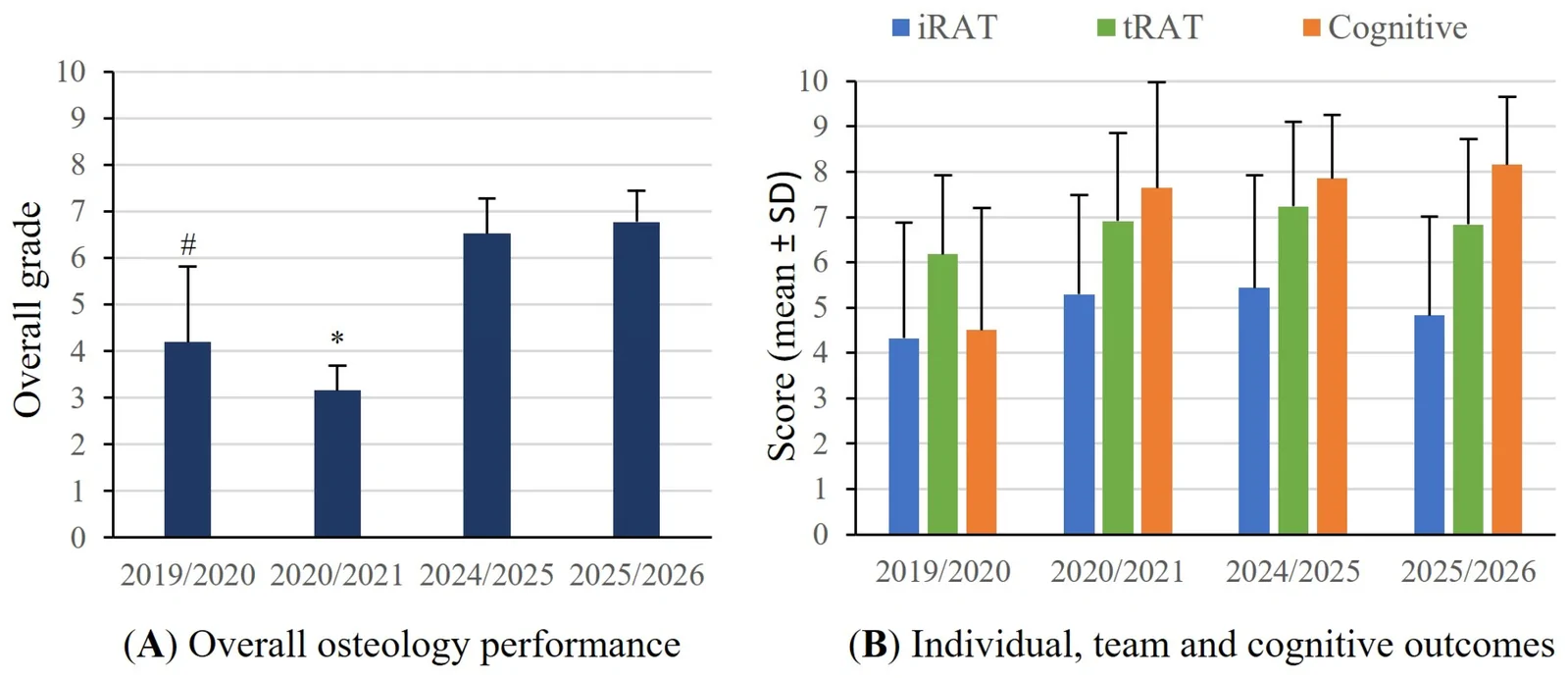
Osteology performance and TBL outcomes across academic years. (**A**) Overall osteology performance (mean ± SD) for students enrolled in the 2019/2020, 2020/2021, 2024/2025, and 2025/2026 academic years. The mean grade for the 2020/2021 academic year was significantly lower than that of all other academic years (* *p* < 0.001). The mean grade for the 2019/2020 academic year was also significantly lower than those obtained in the 2024/2025 and 2025/2026 academic years (# *p* < 0.001) (**B**) Individual Readiness Assurance Test (iRAT), Team Readiness Assurance Test (tRAT), and AOCA scores (mean ± SD) across academic years. All evaluated measures showed significant overall variation among academic years (*p* < 0.001). Post hoc analyses revealed that AOCA scores differed significantly between every pair of academic years (*p* < 0.001). Data are presented as mean ± standard deviation (SD). iRAT, Individual Readiness Assurance Test; tRAT, Team Readiness Assurance Test.

**Figure 4:**
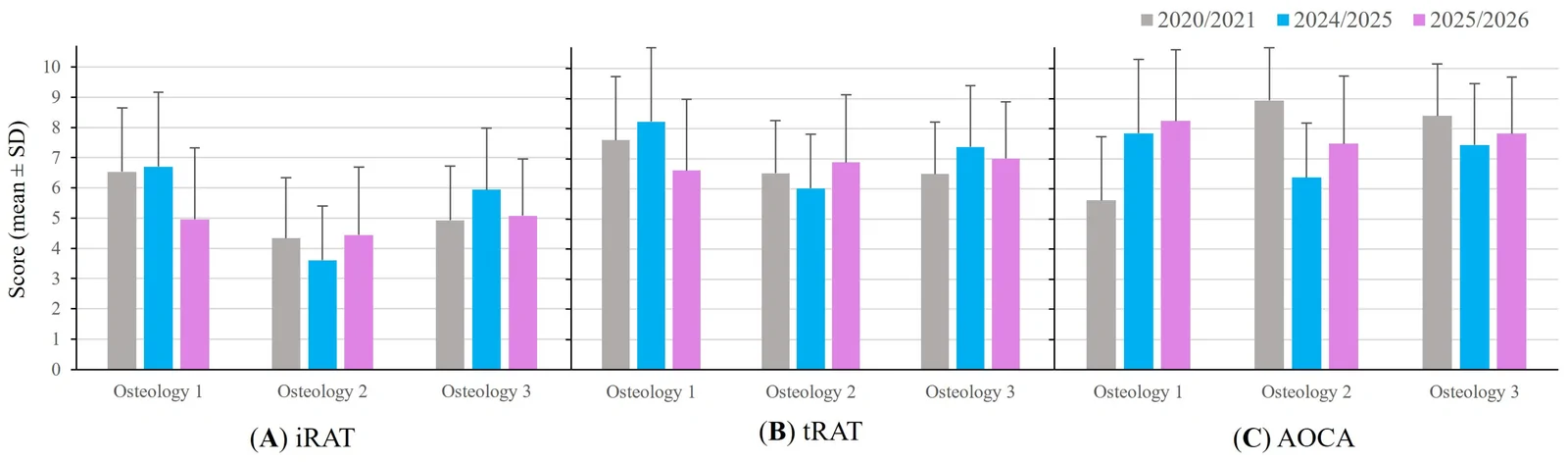
Performance outcomes across osteology practical sessions and academic cohorts.

**Figure 5:**
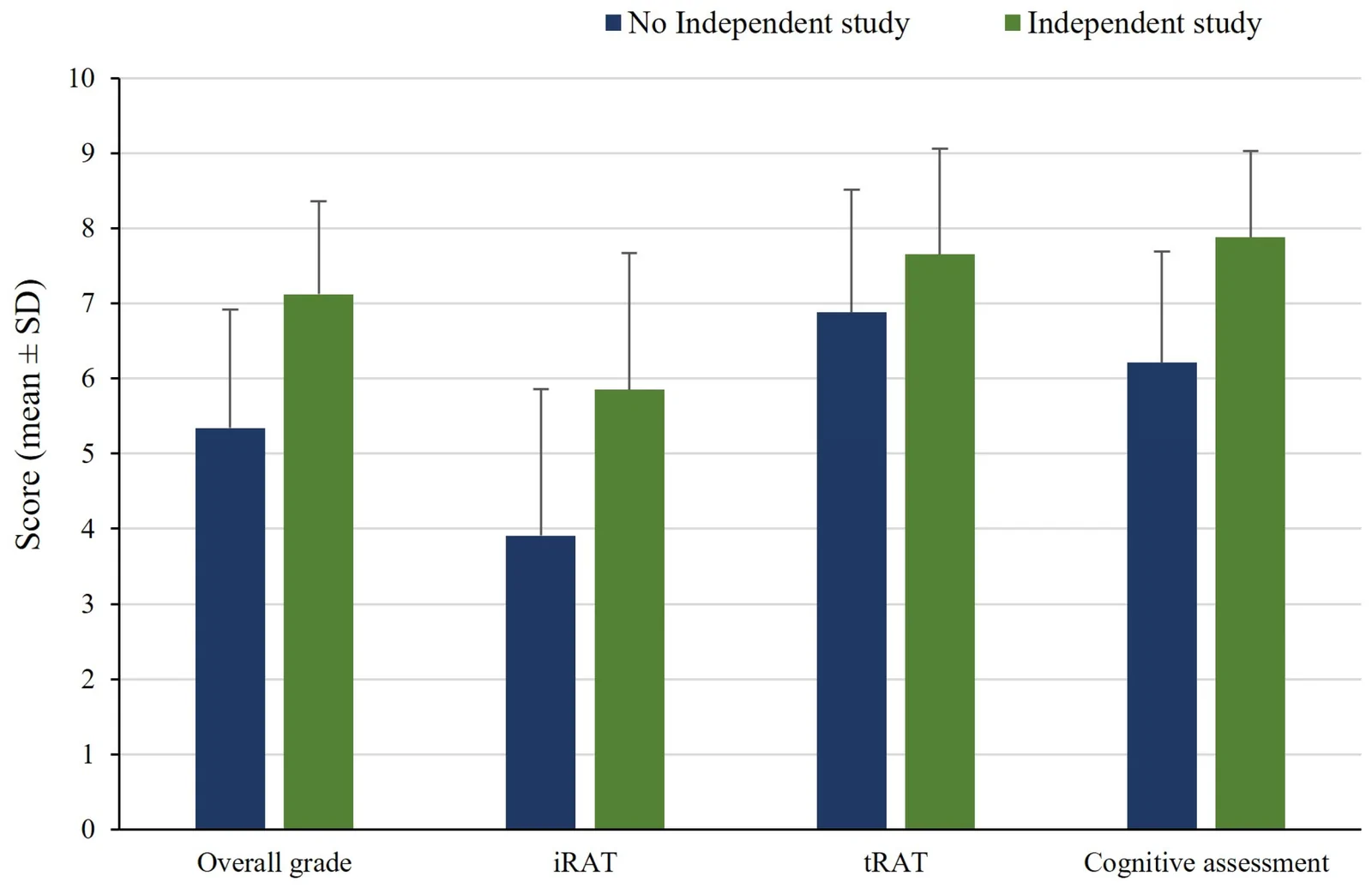
Effect of independent study participation on academic performance.

**Figure 6:**
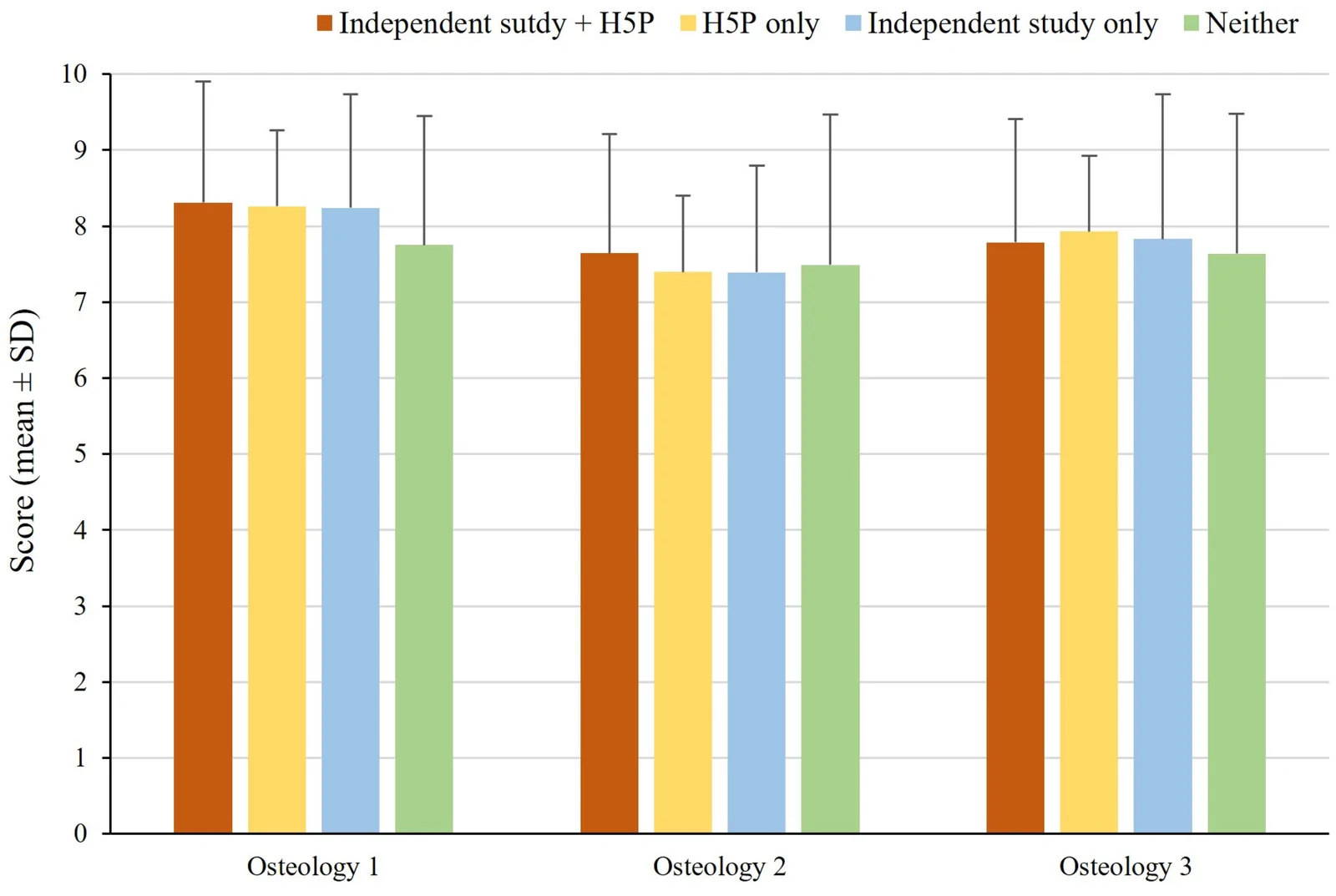
Combined effects of independent study participation and H5P engagement on AOCA performance during osteology practical sessions in the 2025/26 cohort.

**Table 1:** Study Cohorts and Pedagogical Phases.

Cohort	Pedagogical Phase	Teaching Model	*n*
2013/14	Traditional teaching	Demonstration-based practical sessions	184
2014/15	Traditional teaching	Demonstration-based practical sessions	180
2015/16	Incremental pedagogical enhancement	Guided peer discussion and case-based activities	170
2016/17	Incremental pedagogical enhancement	Guided peer discussion and case-based activities	191
2017/18	Incremental pedagogical enhancement	Guided peer discussion and case-based activities	200
2018/19	Incremental pedagogical enhancement	Preparation for TBL implementation	175
2019/20	Full TBL implementation	TBL	155
2020/21	Full TBL implementation	TBL	167
2021/22	Full TBL implementation	TBL	161
2022/23	Full TBL implementation	TBL	208
2023/24	Full TBL implementation	TBL	179
2024/25	Full TBL implementation	TBL	206
2025/26	Full TBL implementation	TBL	169

**Table 2:** Educational resources and student workload across three approaches to anatomy teaching.

Phase	Scheduled Practical Hours		Independent Study	Digital Resources
	Osteology	Sectional anatomy		
Traditional	6	4	none	none
Enhancement	6	4	optional	limited
TBL	6	4	structured	extensive

**Table 3:** Longitudinal Performance in Head Sectional Anatomy Across Pedagogical Phases.

Academic Year	Mean Score	SD	*n*
Traditional Teaching Phase			
2013/14	4.88	2.75	184
2014/15	4.13	2.12	180
Incremental Pedagogical Enhancement Phase			
2015/16	3.11	1.99	170
2016/17	4.29	1.95	191
2017/18	3.31	2.05	200
2018/19	3.21	2.01	175
TBL Phase			
2019/20	4.62	1.75	155
2020/21	4.54	1.35	167
2021/22	5.78	1.39	161
2022/23	6.14	0.84	208
2023/24	5.87	1.06	179
2024/25	6.55	0.97	206
2025/26	6.67	0.95	169

**Table 4:** Osteology Outcomes in Academic Cohorts in Which Team-Based Learning was Implemented.

Academic Year	Overall Grade Mean	SD	iRAT Mean	SD	tRAT Mean	SD	AOCA Mean	SD
Early TBL Implementation								
2019/20	4.19	1.63	4.33	2.54	6.18	1.75	4.51	2.70
2020/21	3.16	0.54	5.30	2.19	6.90	1.94	7.60	2.31
Consolidated TBL Phase								
2024/25	6.52	0.76	5.40	2.49	7.20	1.87	7.90	1.41
2025/26	6.78	0.68	4.80	2.19	6.80	1.89	8.20	1.50

**Table 5:** Effects of Independent Study Participation on Academic Performance.

Outcome Measure	Non-Attendees Mean ± SD	Attendees Mean ± SD	F	*p*	η^2^
Overall Grade	5.34 ± 1.58	7.12 ± 1.24	48.62	<0.001	0.074
iRAT	3.91 ± 1.95	5.85 ± 1.82	62.15	<0.001	0.092
tRAT	6.88 ± 1.63	7.65 ± 1.41	19.34	<0.001	0.031
AOCA	6.21 ± 1.48	7.88 ± 1.15	81.40	<0.001	0.117

**Figure A1:**
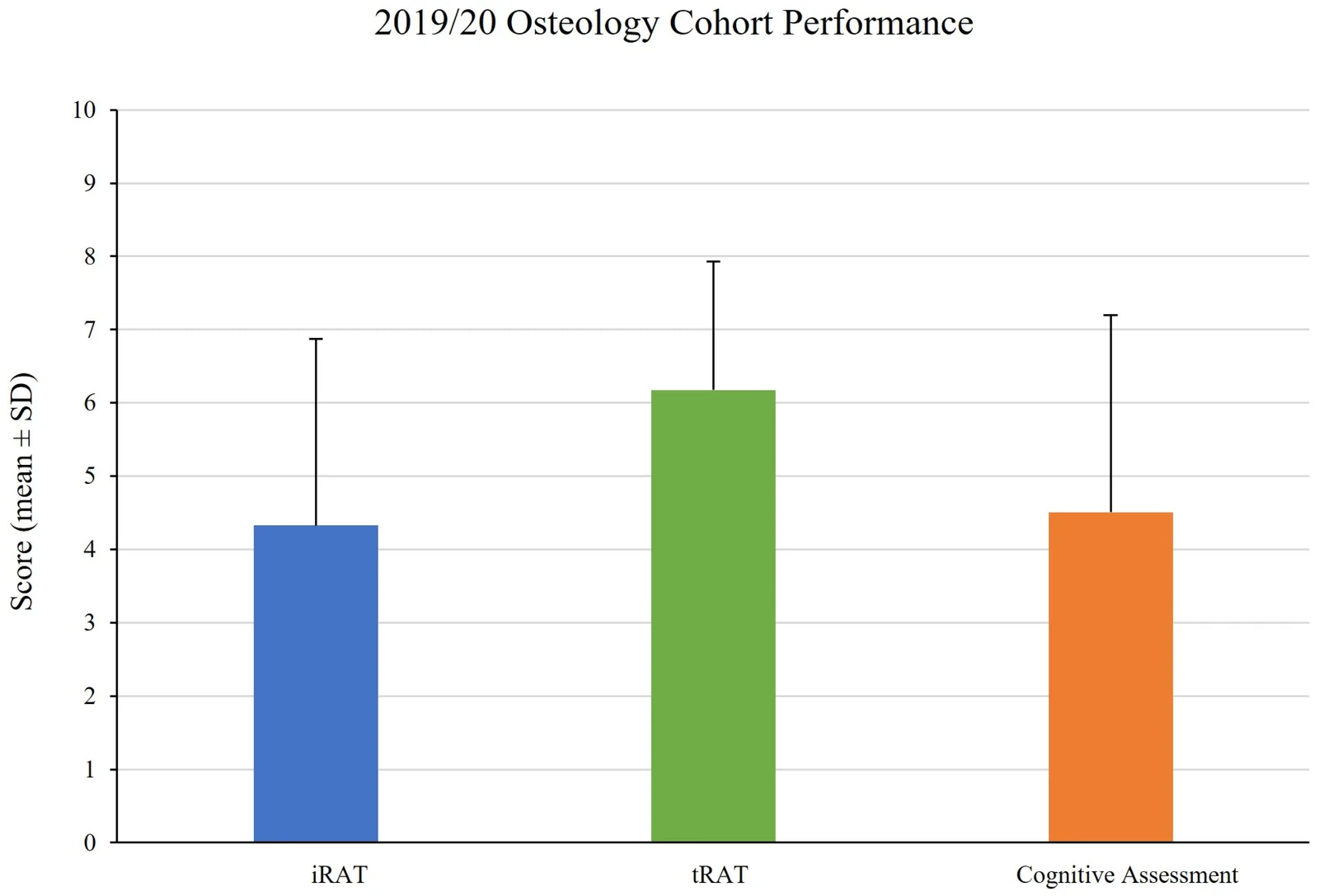
Osteology performance outcomes in the 2019/20 cohort. Bars represent mean ± SD scores for iRAT, tRAT, and AOCA outcomes obtained during the first year of TBL implementation. Team readiness performance exceeded individual readiness performance, whereas AOCA scores remained comparable to individual readiness outcomes.

**Figure A2:**
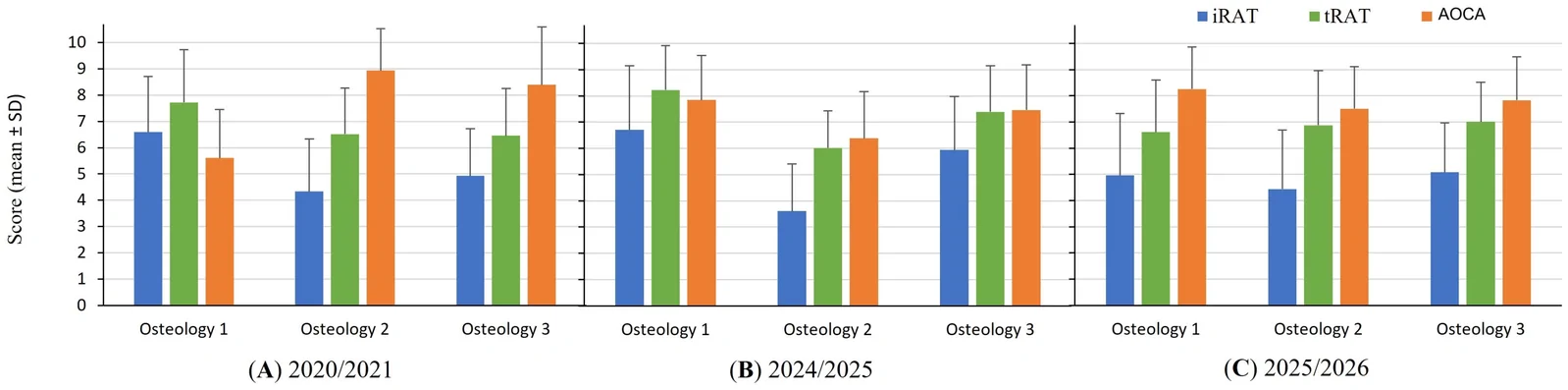
Performance across osteology practical sessions in the 2020/21 (**A**), 2024/25 (**B**), and 2025/26 (**C**) cohorts. Bars represent mean scores ± SD for iRATs, tRATs, and AOCA. Team performance consistently exceeded individual performance across practical sessions. Practice 2 was associated with lower individual achievement in multiple cohorts, whereas AOCA performance remained comparatively high throughout the sequence.

**Figure A3:**
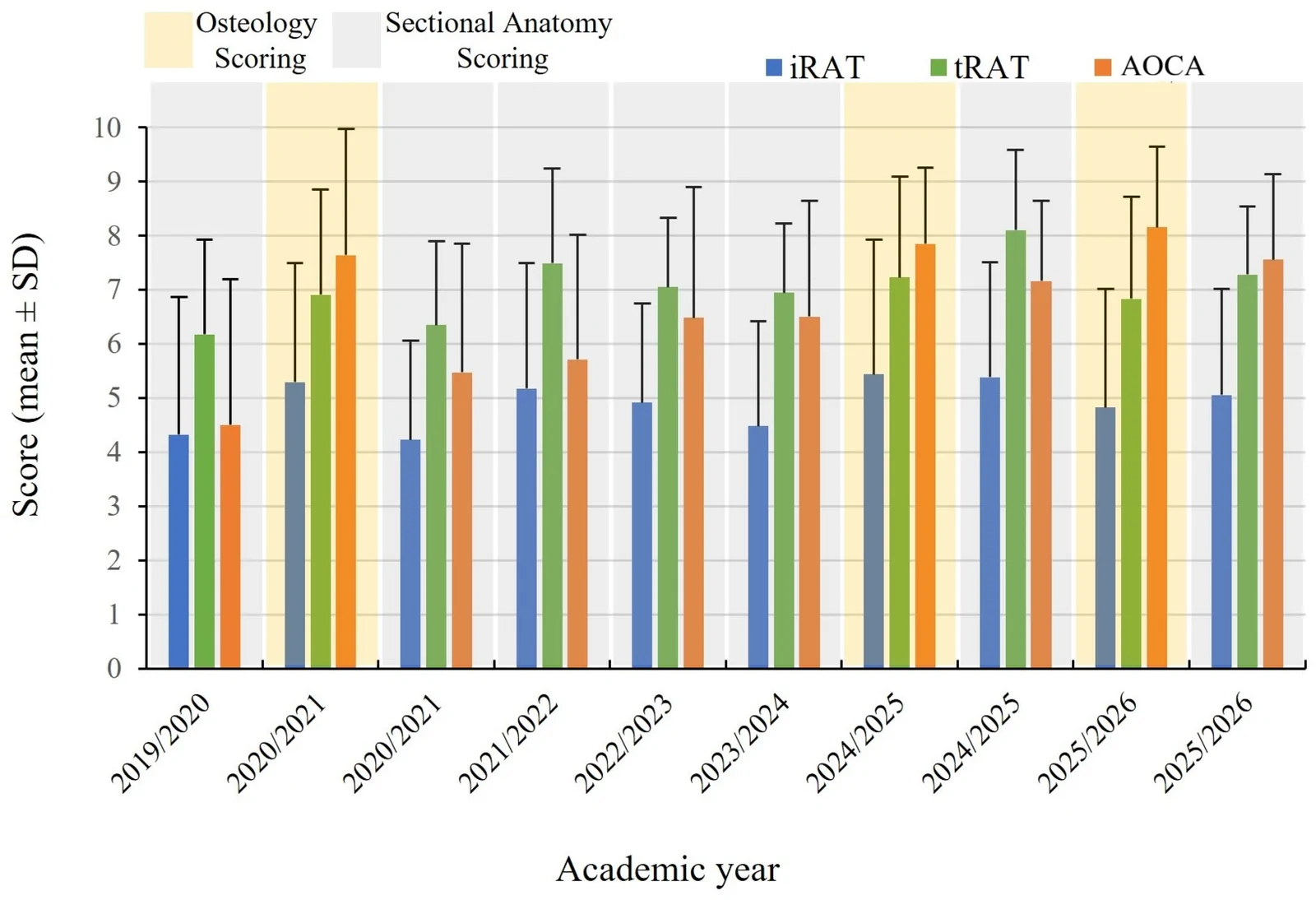
Osteology and sectional anatomy performance across practical sessions in the 2019/20 to 2025/26 cohorts. Bars represent mean ± SD scores for iRAT, tRAT, and AOCA obtained during practical sessions. Yellow-shaded intervals correspond to academic years in which osteology scoring was implemented, whereas gray-shaded intervals indicate the use of sectional anatomy scoring.

**Figure A4:**
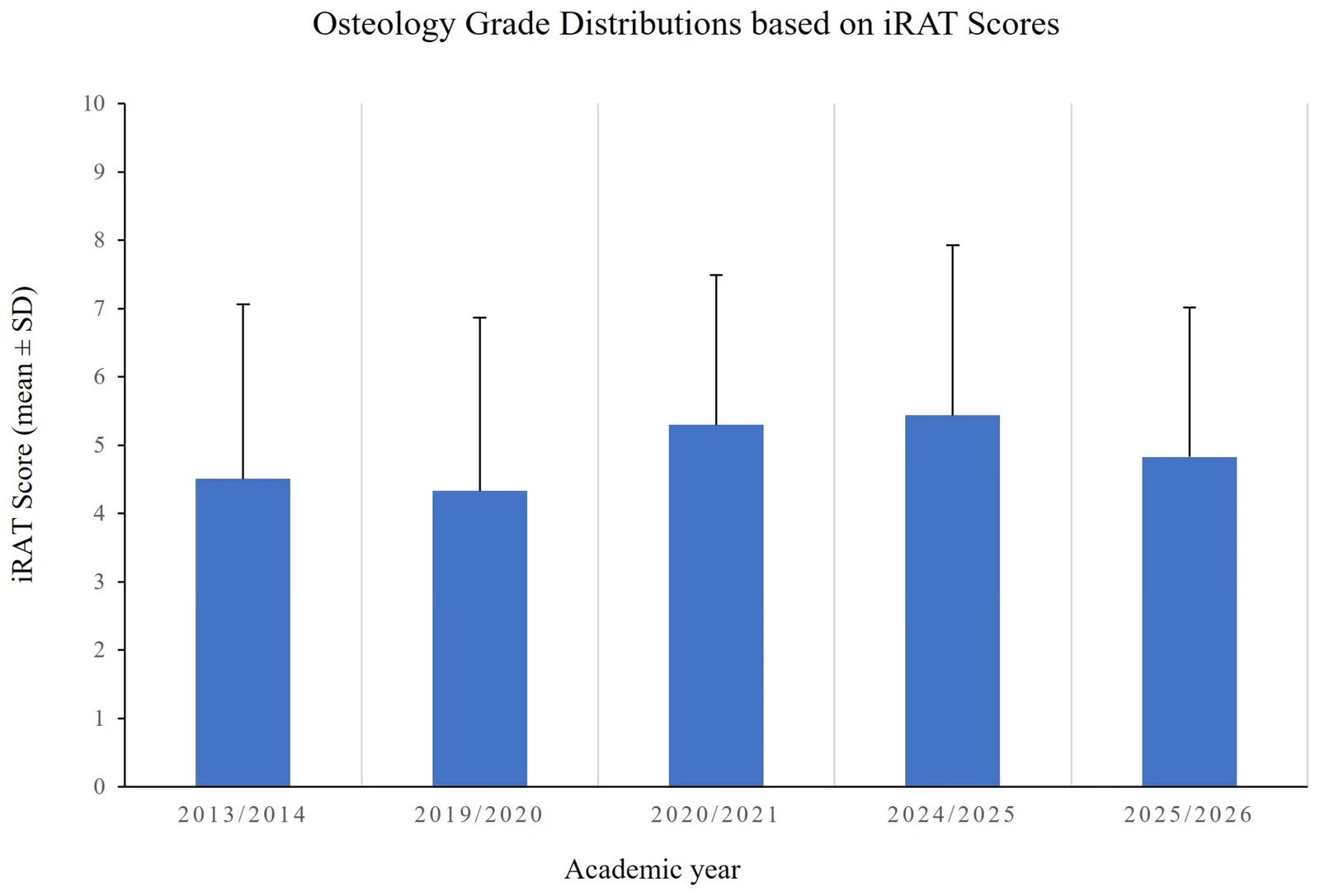
Osteology Grade Distributions Based on iRAT Scores. The longitudinal analysis of osteology grade distributions revealed progressive changes following implementation and consolidation of Team-Based Learning. Mean iRAT scores increased from 4.33 ± 2.54 in the 2019/20 cohort to 5.30 ± 2.19 in 2020/21 and 5.40 ± 2.49 in 2024/25. The most recent cohort (2025/26) achieved a mean score of 4.80 ± 2.19. Although fluctuations were observed between cohorts, the overall pattern suggests improved individual readiness accompanied by more consistent performance during the later stages of curricular implementation.

**Figure A5:**
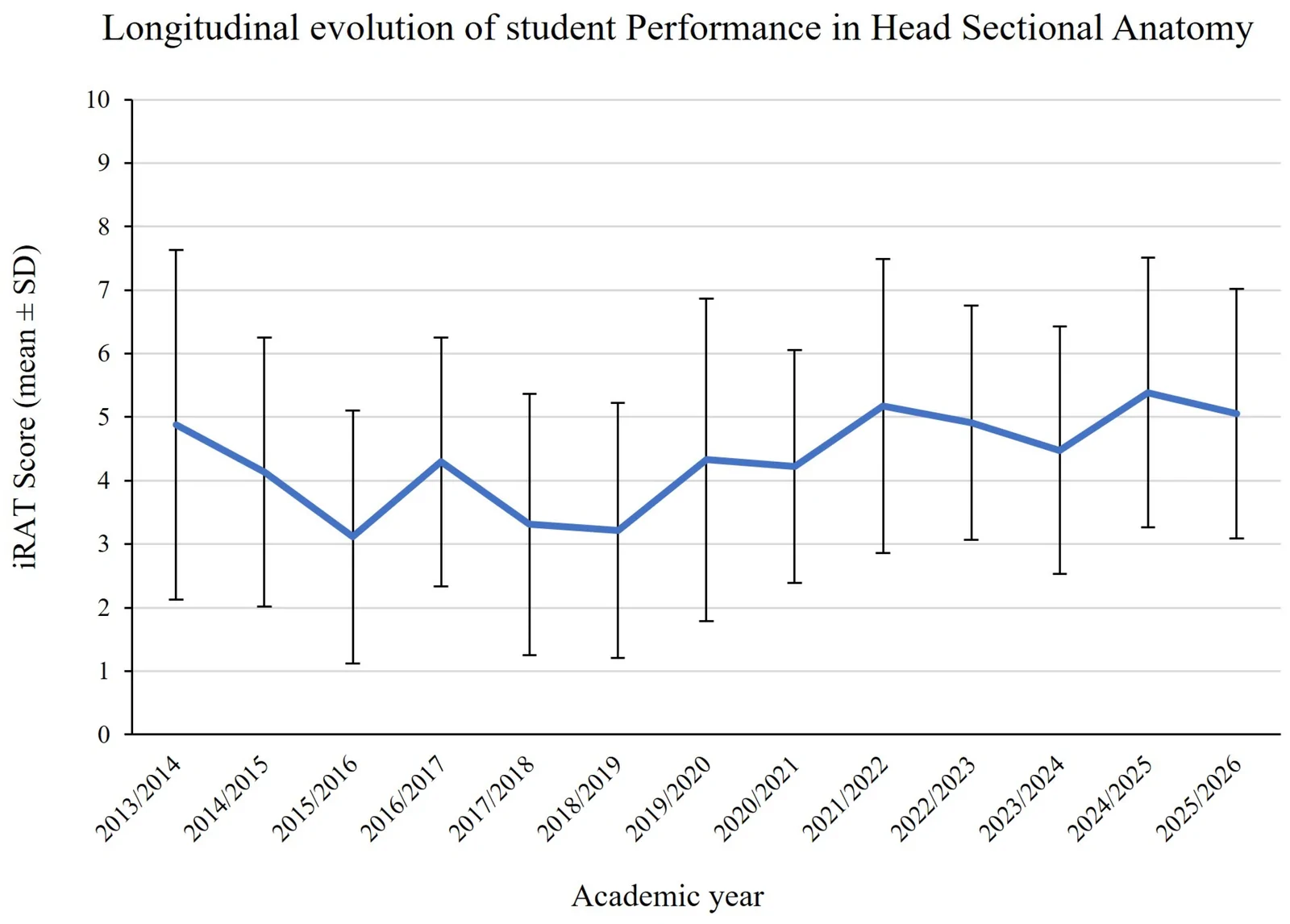
Longitudinal evolution of sectional anatomy grade distributions based on individual readiness assurance (iRAT) scores. Points represent cohort mean iRAT scores and error bars indicate standard deviations (SDs). The dashed vertical line indicates the introduction of Team-Based Learning (TBL) in 2019/20. Mean performance increased progressively following TBL implementation, while variability became more homogeneous in recent cohorts. Values adjacent to each point indicate cohort mean scores.

## Data Availability

The original contributions presented in this study are included in the article. Further inquiries can be directed to the corresponding author.

## Abbreviations

The following abbreviations are used in this manuscript:

AOCA	Application-oriented cognitive assessment
iRAT	Individual readiness assurance test
RAT	Readiness assurance test
TBL	Team-based Learning
tRAT	Team readiness assurance test

## Appendix A

### Longitudinal Performance Across Individual Practical Sessions

This appendix presents detailed cohort-level performance data for iRAT, tRAT, and AOCA across osteology and sectional anatomy practical sessions. These supplementary analyses provide the practical-level information underlying the longitudinal trends reported in Figure 2, Figure 3 and Figure 4 and allow detailed examination of the evolution of learning outcomes throughout the implementation of TBL.

**Table A1:** Osteology performance.

Academic Year	Practical Session	iRAT	tRAT	AOCA
	Osteology 1	6.53 ± 2.10	7.62 ± 2.12	5.62 ± 1.85
2020/21	Osteology 2	4.34 ± 2.00	6.52 ± 1.75	8.34 ± 1.37
	Osteology 3	4.33 ± 1.80	6.50 ± 1.73	8.43 ± 2.10
	Osteology 1	6.70 ± 2.46	8.23 ± 1.69	7.80 ± 1.31
2024/25	Osteology 2	3.60 ± 1.80	6.01 ± 1.42	6.40 ± 1.36
	Osteology 3	5.94 ± 2.03	7.40 ± 1.76	7.50 ± 1.23
	Osteology 1	4.96 ± 2.37	6.61 ± 2.00	8.30 ± 1.24
2025/26	Osteology 2	4.44 ± 2.25	6.88 ± 2.09	7.50 ± 1.61
	Osteology 3	5.08 ± 1.89	7.01 ± 1.52	7.80 ± 1.26

## Appendix B

Grade distribution across curricular transformation. This appendix presents the longitudinal distribution of grades obtained in osteology and sectional anatomy practical sessions throughout the study period. The purpose of these supplementary analyses is to provide additional evidence regarding the evolution of student achievement and score variability during the transition from traditional practical teaching to TBL. The analyses focus on iRAT, allowing direct comparison across cohorts because this measure was available throughout the study period. Figure A4 presents osteology grade distributions and Figure A5 presents sectional anatomy grade distributions based on iRAT scores. Together, these supplementary analyses provide additional evidence regarding the evolution of student achievement and score variability throughout the implementation of TBL.

### Appendix B.1. Osteology Grade Distributions

Figure A4 illustrates the distribution of osteology performance based on iRAT scores across cohorts for which comparable individual readiness data were available. These distributions provide additional context for the longitudinal improvements reported in the main manuscript by demonstrating changes in both average achievement and score dispersion over time.

### Appendix B.2. Sectional Anatomy Grade Distribution

Figure A5 illustrates the distribution of sectional anatomy performance across academic cohorts using iRAT scores as the common metric for comparison. These results complement Figure 1 and provide additional information regarding changes in score distributions throughout the curricular transformation.

Figure A5 complements the longitudinal results presented in Figure 1 by illustrating changes in sectional anatomy grade distributions throughout the curricular transformation. The figure shows substantial variability during the traditional and transitional teaching periods, followed by a progressive increase in mean achievement after implementation of TBL. The highest mean scores were observed in the most recent cohorts, supporting the conclusion that the curricular transformation was associated with improved anatomical learning outcomes.

Table 3 11 ± 1.99 to 4.88 ± 2.75 during the pre-TBL period. Following introduction of Team-Based Learning, average scores generally increased, reaching 5.39 ± 2.12 in 2024/25 and 5.06 ± 1.96 in 2025/26. The reduction in score dispersion observed in recent cohorts provides additional evidence for increased consistency in student achievement.

## Appendix C

The 2024/25 cohort demonstrated high levels of academic achievement following consolidation of the Team-Based Learning methodology. Mean overall osteology grades exceeded 6.3 points, whereas team readiness performance consistently surpassed individual readiness performance. AOCA scores remained high throughout the practical sequence, although a temporary decline was observed during Practice 2. Students participating in independent study activities generally achieved higher performance outcomes than non-participants. Furthermore, sectional anatomy achievement increased progressively across osteology grade categories, supporting the integration of osteological and spatial anatomical learning outcomes within the curriculum.

### Cohort 2024/25: Detailed Performance Dataset

This appendix provides a detailed summary of the 2024/25 cohort, including overall osteology performance, practical-session outcomes, independent study analyses, and the relationship between osteology achievement and sectional anatomy performance. These supplementary data support the analyses presented in Figure 2, Figure 3 and Figure 4 of the main manuscript.

**Table A2:** Osteology Performance Outcomes (2024/25).

Variable	Mean	SD	Min	Max	*n*
Overall Grade	6.52	0.76	0.90	7.88	206
iRAT	5.40	2.49	0.00	10.00	206
tRAT	7.23	1.87	3.00	10.00	206
AOCA	7.90	1.41	2.00	10.00	206

Table A2 shows the descriptive statistics for osteology performance outcomes obtained in the 2024/25 cohort. Values are presented as mean, standard deviation (SD), minimum score (Min), maximum score (Max), and sample size (n).

**Table A3:** Performance by Practical Session (2024/25).

Variable	Osteology 1	Osteology 2	Osteology 3
iRAT (Mean ± SD)	6.70 ± 2.46	3.60 ± 1.80	5.94 ± 2.03
tRAT (Mean ± SD)	8.23 ± 1.69	6.01 ± 1.42	7.40 ± 1.76
AOCA (Mean ± SD)	7.80 ± 1.31	6.40 ± 1.36	7.50 ± 1.23

Table A3 shows the osteology performance by practical session in the 2024/25 cohort. Values are presented as mean ± SD. Osteology 2 was consistently associated with lower performance across readiness and AOCA domains, followed by recovery during Osteology 3.

**Table A4:** Independent Study Analysis (2024/25).

Outcome Measure	No Study Mean ± SD	Study Mean ± SD
Overall Grade	6.21 ± 1.48	6.78 ± 1.32
iRAT	4.98 ± 2.37	6.04 ± 2.50
tRAT	7.13 ± 1.79	7.50 ± 1.92
AOCA	7.26 ± 1.10	7.29 ± 0.93

iRAT = Individual Readiness Assurance; SD = standard deviation; tRAT = Team Readiness Assurance.

Table A4 shows the comparison of performance outcomes according to participation in independent study activities during the 2024/25 academic year. Students attending independent study sessions generally achieved higher scores across readiness and overall performance measures.

**Table A5:** Relationship Between Osteology Achievement and Sectional Anatomy Performance.

Osteology Grade Category	Sectional Anatomy Mean ± SD
<6.50	4.04 ± 2.02
6.50–7.02	4.37 ± 1.92
>7.02	5.13 ± 2.23

SD = standard deviation.

Table A5 shows the sectional anatomy performance according to osteology achievement category in the 2024/25 cohort. Students obtaining the highest osteology grades also achieved the highest sectional anatomy scores, suggesting a positive association between osteological competence and sectional anatomy performance.

## Appendix D

This appendix provides a detailed summary of academic performance outcomes for the 2025/26 cohort, representing the most recent stage of Team-Based Learning (TBL) implementation. The appendix includes overall osteology outcomes, practical-session analyses, the combined effects of independent study participation and H5P engagement, and comparisons with the preceding academic cohort. These supplementary data support the analyses presented in Figure 2, Figure 3, Figure 4 and Figure 5 of the main manuscript.

The 2025/26 cohort represents the most advanced stage of TBL implementation evaluated in this study. Overall grades reached their highest values of the study period, while AOCA scores exceeded 8.0 points on average. Compared with earlier cohorts, performance remained comparatively stable across practical sessions, suggesting increased adaptation of students to the TBL format and greater consistency of learning outcomes.

AOCA outcomes remained high regardless of supplementary learning strategy. Students engaging with independent study activities, H5P resources, or both achieved very similar performance levels, while students relying exclusively on standard instructional materials also maintained satisfactory achievement. These findings support the interpretation that independent study activities and digital resources provide additional educational benefits beyond an already effective instructional foundation.

Comparison with the 2024/25 cohort indicated improvements in individual readiness performance and practical assessment outcomes while maintaining strong collaborative learning performance. Collectively, these findings suggest that continued refinement of the TBL methodology contributed to increasingly stable and effective learning outcomes over time.

**Table A6:** Osteology Performance Outcomes (2025/26).

Variable	Mean	SD	Minimum	Maximum	*n*
Overall Grade	6.78	0.68	4.45	8.09	169
iRAT	4.80	2.19	0.00	10.00	169
tRAT	6.80	1.89	2.00	10.00	169
AOCA	8.20	1.50	NR	10.00	169

Note. Values are presented as descriptive statistics for the 2025/26 cohort.

**Table A7:** Performance Across Osteology Practical Sessions (2025/26).

Outcome Measure	Practice 1	Practice 2	Practice 3
iRAT	4.96 ± 2.37	4.44 ± 2.25	5.08 ± 1.89
tRAT	6.61 ± 2.00	6.88 ± 2.09	7.01 ± 1.52
AOCA	8.30 ± 1.24	7.50 ± 1.61	7.80 ± 1.26

**Note.** Values are presented as mean ± SD.

**Table A8:** Independent Study and H5P Resource Engagement.

Learning Strategy	Practice 1 Mean ± SD	Practice 2 Mean ± SD	Practice 3 Mean ± SD
Independent Study + H5P	8.30 ± 1.07	7.60 ± 1.55	7.80 ± 1.28
H5P Only	8.30 ± 1.12	7.40 ± 1.46	7.90 ± 1.36
Independent Study Only	8.20 ± 1.17	7.40 ± 1.43	7.80 ± 1.50
Standard Materials Only †	7.80 ± 1.22	7.50 ± 1.75	7.60 ± 1.72

† Students in this category did not attend independent study sessions and did not use H5P resources but prepared practical sessions using the PowerPoint-based study materials routinely provided for all students. **Note.** Results are descriptive because individual-level data were not available for formal inferential analyses among groups. SD = standard deviation.

## References

[1] Lignereux Y. (1993). L’enseignement de l’anatomie, de l’histologie et de l’embryologie vétérinaires. Une enquête mondiale: Rapport, propositions. Rev. Méd. Vét..

[2] Provo J.A., Lamar C.H. (1995). Prosection as an approach to student-centered learning in veterinary gross anatomy. J. Am. Vet. Med. Assoc..

[3] Provo J.A., Lamar C.H., Bill R.L. (1996). Increasing student-centered learning opportunities in a veterinary comparative anatomy course. J. Vet. Med. Educ..

[4] Gonçalves E., Capucha L. (2020). Student-centered and ICT-enabled learning models in veterinarian programs: What changed with COVID-19?. Educ. Sci..

[5] Gordon S., Bolwell C., Raney J.L., Zepke N. (2022). Transforming a didactic lecture into a student-centered active learning exercise—Teaching equine diarrhea to fourth-year veterinary students. Educ. Sci..

[6] Escudero A., Simó-Martínez M.S., Morera M., Navarro-Serra A., García-Manzanares M. (2025). Active learning in veterinary anatomy education: Investigating the impact of peer-led Q&A games and multimedia on student perceptions. Vet. Sci..

[7] Martín-Alguacil N., Mota-Blanco R., Avedillo L., Marañón-Almendros M., Gallego-Agúndez M. (2025). Timing, tools, and thinking: H5P-driven engagement in flipped veterinary education. Vet. Sci..

[8] Hazel S., Heberle N., McEwen M., Adams K. (2013). Team-based learning increases active engagement and enhances development of teamwork and communication skills in a first-year course for veterinary and animal science undergraduates. J. Vet. Med. Educ..

[9] Ormandy E. (2021). Team-based learning within the veterinary curriculum. Dev. Acad. Pract..

[10] Michaelsen L.K., Parmelee D.X., McMahon K.K., Levine R.E. (2007). Team-Based Learning for Health Professions Education: A Guide to Using Small Groups for Improving Learning.

[11] Vasan N.S., DeFouw D.O., Compton S. (2011). Team-based learning in anatomy: An efficient, effective, and economical strategy. Anat. Sci. Educ..

[12] Al-Neklawy A.F., Ismail A.S.A. (2022). Online anatomy team-based learning using Blackboard Collaborate platform during the COVID-19 pandemic. Clin. Anat..

[13] Hashmi N.R. (2014). Team based learning (TBL) in undergraduate medical education. J. Coll. Physicians Surg. Pak..

[14] Sterpu I., Herling L., Nordquist J., Rotgans J.I., Acharya G. (2024). Team-based learning (TBL) in clinical disciplines for undergraduate medical students—A scoping review. BMC Med. Educ..

[15] Evans J. (1987). Evolution of veterinary anatomy teaching. Schweiz. Arch. Tierheilkd..

[16] Varner C., Dixon L., Simons M.C. (2021). The past, present, and future: A discussion of cadaver use in medical and veterinary education. Front. Vet. Sci..

[17] Clement S.M., Ubben T.A., Yates D.T. (2024). Cadaveric prosections prepared by qualified instructional staff were more efficient and effective teaching modalities for veterinary gross anatomy than in-class dissections by students. J. Vet. Med. Educ..

[18] Cake M.A. (2006). Deep dissection: Motivating students beyond rote learning in veterinary anatomy. J. Vet. Med. Educ..

[19] Jansen M., Cavdar I.K. (2025). Adapting team-based learning for medical education: A case study with scalable and resource-efficient implementation. Med. Sci. Educ..

[20] Martín-Alguacil N., Avedillo L.J. (2024). Student-centered active learning improves performance in solving higher-level cognitive questions in health sciences. Int. Med. Educ..

[21] Parmelee D., Michaelsen L.K., Cook S., Hudes P.D. (2012). Team-based learning: A practical guide: AMEE Guide No. 65. Med. Teach..

[22] Martín-Alguacil N., Avedillo L. (2026). From illusion of competence to productive uncertainty: A longitudinal mixed-methods study of team-based learning in anatomy education. Educ. Sci..

[23] Alizadeh M., Masoomi R., Mafinejad M.K., Parmelee D.X., Khalaf R.J., Norouzi A. (2024). Team-based learning in health professions education: An umbrella review. BMC Med. Educ..

[24] Roossien L., Van Klaveren L.-M., De Vos R., Dolmans D., Boerboom T.B.B. (2025). Team-based learning (TBL) in health professions education: A systematic review on its conceptual elements and outcomes. MEDTEACH.

[25] Koles P.G., Stolfi A., Borges N.J., Nelson S., Parmelee D.X. (2010). The impact of team-based learning on medical students’ academic performance. Acad. Med..

[26] Tan N.C.K., Kandiah N., Chan Y.H., Umapathi T., Lee S.H., Tan K. (2011). A controlled study of team-based learning for undergraduate clinical neurology education. BMC Med. Educ..

[27] Burgess A., van Diggele C., Roberts C., Mellis C. (2020). Team-based learning: Design, facilitation and participation. BMC Med. Educ..

[28] Xie Z.B., Cheng X.Y., Li X.Y., Zhang Y.F. (2025). Team-based learning pedagogy enhances the education quality: A systematic review and meta-analysis. BMC Med. Educ..

[29] Martín-Alguacil N., Avedillo L.J., Gallego-Agúndez M., Mota-Blanco R. (2024). Student-centered learning: Some issues and recommendations for its implementation in a traditional curriculum setting in health sciences. Educ. Sci..

[30] Nurhadi A.A., Jones L., Budu (2025). Team up for better learning: Evaluating team-based learning in anatomy education. Int. J. Eval. Res. Educ..

[31] Martín-Alguacil N., Avedillo L., Mota-Blanco R.A., Marañón-Almendros M., Gallego-Agúndez M. (2025). ChatGPT as a virtual peer: Enhancing critical thinking in flipped veterinary anatomy education. Int. Med. Educ..

[32] Hrynchak P., Batty H. (2012). The educational theory basis of team-based learning. Med. Teach..

[33] Roach V.A., Mi M., Mussell J.C., Lufler R.S., DeVeau K.M., Dunham S., Husmann P.R., Herriott H.L., Edwards D.N., Doubleday A.F. (2020). Correlating spatial ability with anatomy assessment performance: A meta-analysis. Anat. Sci. Educ..

[34] Rahmani M.Z., Bukhari A., Wiyono N., Amru K., Baharuddin B., Nurhadi A.A. (2024). Exploring links between visuospatial ability and anatomy learning in education: A bibliometric analysis and scientific mapping. Narra J..

[35] Berney S., Bétrancourt M., Molinari G., Hoyek N. (2015). How spatial abilities and dynamic visualizations interplay when learning functional anatomy with 3D anatomical models. Anat. Sci. Educ..

[36] Bogomolova K., Hierck B., Hage J.A., Hovius S. (2020). Anatomy Dissection Course Improves the Initially Lower Levels of Visual-Spatial Abilities of Medical Undergraduates. Anat. Sci. Educ..

[37] Nokovitch L., Deneuve S., DeAndrade V., Gagnayre R., Margat A. (2025). Visual–spatial abilities enhancement and spatial anatomy learning: A systematic review. Med. Educ..

[38] Sagoo M., Lam P.Y., Theivendran S., Wingate R. (2025). Exploring the potential malleability of spatial skills through anatomy teaching: A quantitative study among medical students. Anat. Sci. Educ..

[39] Ngoc P., Cheng C.-L., Lin Y.-K., Wu M., Chu J.-S., Tang K.-P. (2020). A meta-analysis of students’ readiness assurance test performance with team-based learning. BMC Med. Educ..

[40] Jabeen K., Rehman J., Fatima S.S., Ahmed I., Ghias K. (2026). Does medical students’ perception of team-based learning have an impact on achieving planned learning outcomes?. BMC Med. Educ..

[41] Dörrenbächer-Ulrich L., Gerics B., Steinberg E. (2025). Self-Regulated Learning in Early-Year Veterinary Students: Analyzing Strategy Usage and Strategy Knowledge in Anatomy Classes. J. Vet. Med. Educ..

[42] Akhtar S., Channon S., Previdelli R. (2026). Digital Learning Interactions in Veterinary Anatomy Education Enhance Self-Directed Learning and Student Satisfaction. J. Vet. Med. Educ..

[43] Frame T.R., Cailor S.M., Gryka R., Chen A.M.H., Kiersma M.E., Sheppard L. (2015). Student Perceptions of Team-based Learning vs Traditional Lecture-based Learning. Am. J. Pharm. Educ..

[44] Andrews-Dickert R., Nagaraj R., Zhan L., Knittig L., Zhao Y. (2024). Improving learning experience through implementing standardized team-based learning process in undergraduate medical education. BMC Med. Educ..

